# Effect of psilocybin therapy on suicidal ideation, attempts, and deaths in people with psychiatric diagnoses: a systematic review and meta-analysis

**DOI:** 10.1177/20451253251372449

**Published:** 2025-09-07

**Authors:** Stanley Wong, Gray Meckling, Nicholas Fabiano, Sanghun Lee, Brett D. M. Jones, Risa Shorr, Aroldo Dargel, Alan K. Davis, Jess G. Fiedorowicz, Marco Solmi, Joshua D. Rosenblat, Benoit H. Mulsant, Daniel M. Blumberger, Muhammad Ishrat Husain

**Affiliations:** Department of Psychiatry, University of Toronto, 250 College Street, Toronto, ON M5T 1R8, Canada; Department of Psychiatry, University of Ottawa, Ottawa, ON, Canada; Department of Psychiatry, University of Ottawa, Ottawa, ON, Canada; Department of Psychiatry, University of Toronto, Toronto, ON, Canada; Department of Psychiatry, University of Toronto, Toronto, ON, Canada; Campbell Family Mental Health Research Institute, Centre for Addiction and Mental Health, Toronto, ON, Canada; Library Services, The Ottawa Hospital, Ottawa, ON, Canada; Department of Psychiatry, University of Ottawa, Ottawa, ON, Canada; Department of Mental Health, The Ottawa Hospital, Ottawa, ON, Canada; Ottawa Hospital Research Institute (OHRI), Ottawa, ON, Canada; Center for Psychedelic Drug Research and Education, College of Social Work, The Ohio State University, Columbus, OH, USA; Center for Psychedelic and Consciousness Research, School of Medicine, Johns Hopkins University, Baltimore, MD, USA; Department of Internal Medicine, Ohio State University, Columbus, OH, USA; Department of Psychiatry, University of Ottawa, Ottawa, ON, Canada; Department of Mental Health, The Ottawa Hospital, Ottawa, ON, Canada; Ottawa Hospital Research Institute (OHRI), Ottawa, ON, Canada; School of Epidemiology and Public Health, Faculty of Medicine, University of Ottawa, Ottawa, ON, Canada; Department of Psychiatry, University of Ottawa, Ottawa, ON, Canada; Department of Mental Health, The Ottawa Hospital, Ottawa, ON, Canada; Ottawa Hospital Research Institute (OHRI), Ottawa, ON, Canada; School of Epidemiology and Public Health, Faculty of Medicine, University of Ottawa, Ottawa, ON, Canada; Department of Child and Adolescent Psychiatry, Charité Universitätsmedizin, Berlin, Germany; Department of Psychiatry, University of Toronto, Toronto, ON, Canada; Mood Disorder Pharmacology Unit, University Health Network, Toronto, ON, Canada; Department of Psychiatry, University of Toronto, Toronto, ON, Canada; Campbell Family Mental Health Research Institute, Centre for Addiction and Mental Health, Toronto, ON, Canada; Department of Psychiatry, University of Toronto, Toronto, ON, Canada; Campbell Family Mental Health Research Institute, Centre for Addiction and Mental Health, Toronto, ON, Canada; Temerty Centre for Therapeutic Brain Intervention, CAMH, Toronto, ON, Canada; Department of Psychiatry, University of Toronto, Toronto, ON, Canada; Campbell Family Mental Health Research Institute, Centre for Addiction and Mental Health, Toronto, ON, Canada

**Keywords:** psilocybin, psychedelics, suicidal ideation, suicide, suicide attempts

## Abstract

**Background:**

Suicidal ideation, attempts, and deaths present a major and tragic public health concern. Recent trials of psilocybin therapy (PT) have shown promise in treating treatment-resistant depression and have found a reduction in suicidal ideation. Given the growth of PT research, there is a need to further understand its effect on suicidal ideation, attempts, and deaths.

**Objective:**

To assess and synthesize evidence on the effects of PT on suicidal ideation, attempts, and deaths in psychiatric patients.

**Design:**

PRISMA-compliant systematic review and meta-analysis.

**Data source:**

MEDLINE, EMBASE, Cochrane, and PsychINFO.

**Method:**

Databases were searched for randomized controlled trials of PT in adults with psychiatric diagnoses that reported suicide outcomes (ideation, attempts, and deaths). Abstract and full-text screening were conducted, and suicide outcomes were extracted. Meta-analysis was performed with a random effects model to assess changes in suicide outcomes compared to control through the standardized mean difference (SMD). Assessment of heterogeneity, risk of bias, and subgroup analysis was completed.

**Results:**

Nine studies were included (*N* = 593; 335 psilocybin & 258 control). Two studies were excluded from meta-analysis because suicide-related outcomes data were not available. Participants with PT experienced a small and significant decrease in suicidal ideation compared to control (*k* = 7, SMD = −0.24, 95% CI −0.42 to −0.06, *p* = 0.008, *I*^2^ = 0%). There was no publication bias found. Subgroup analysis found no significant differences between groups. No study reported suicide attempts or suicide deaths. Two studies had a high risk of bias.

**Conclusion:**

Psilocybin therapy may reduce suicidal ideation in adults with psychiatric diagnoses. Current studies are limited by small sample size, lack of follow-up data, and assessment of blinding.

**Trial registration:**

CRD42023445706.

## Plain language summary

The effects of psilocybin on suicidal ideation, attempts, and deaths

1. This systematic review and meta-analysis evaluated the effects of psilocybin therapy (PT) suicidal ideations, attempts, and deaths in people living with a psychiatric disorder.

2. Nine studies involved 593 adults, comparing those who received PT (335 people) to those who did not and were in a control group (258 people).

3. PT led to a small but significant reduction in suicidal ideation. There were no instances of suicide attempts or completed suicides reported. However, most studies did not report on suicide attempts or completed suicides.

4. Current studies had limitations, such as low number of participants, lack of long-term follow-up, and issues with how the participants were blinded in a study.

5. PT may help reduce suicidal ideation in people with a psychiatric disorder, but more higher quality research is needed to confirm these findings.

## Introduction

Suicide accounts for over 800,000 deaths annually worldwide. Accounting for 1.4% of all annual deaths globally, it is a top 10 leading cause of death in many countries and is the fourth leading cause of death among people aged 15–29.^1^ Over a 27-year period, deaths by suicide have increased by 6.7% and suicide is recognized as a major public health issue.^1^ Suicidal ideation and attempts are reported to be associated with suicide deaths.^2^ In the United States, 13.2 million adults have had serious thoughts about suicide, 3.8 million made plans for suicide, and 1.6 million attempted suicide in 2022.^3^ There are also economic implications, contributing to over $93 billion in medical costs and lost productivity from hospitalizations and disability.^4^ Suicidal ideation (SI), suicide attempts (SA), and suicide deaths are influenced by various factors, including mental health conditions, social and economic disparities, access to healthcare, and traumatic life events.^5^ The progression from SI to SA is influenced by dispositional, acquired, and practical contributors.^6^ Given the urgency and gravity of the suicide crisis, researchers have been exploring new approaches to treatment and prevention.

Reducing suicide outcomes is a critical goal in mental health care, and several treatments have been identified as promising approaches. One of the most well-established interventions is psychotherapy through cognitive behavior therapy (CBT) and dialectical behavior therapy (DBT)^7^. CBT has shown evidence in reducing SI in veterans and preventing follow-up SA in people with SI or post-SA.^8–10^ Furthermore, there is also evidence of neurostimulation, such as electroconvulsive therapy, in reducing suicide deaths in people with depression.^11^

Pharmacological interventions, including antipsychotics, lithium, and ketamine, have shown some evidence of potential protective effects against suicide.^12–16^ However, the mechanism for these classes of medication is not fully understood. It is believed that antipsychotics target psychotic and depressive symptoms, which may contribute to their effectiveness in suicide prevention.^15^ Lithium is believed to reduce impulsive and aggressive behavior that may lead to increased SI and SA.^16^ Ketamine is thought to involve the modulation of glutamate transmission and the promotion of synaptic plasticity.^17^ Despite these various treatments, there is no gold standard of treatment for SI, SA, and suicide deaths.^18^

Recently, there has been a resurging interest in the use of psychedelics combined with psychological support to treat several psychiatric and substance use disorders. Psilocybin is a psychoactive tryptamine alkaloid found in *Psilocybe* genus species of mushrooms.^19^ Psilocybin therapy (PT) has shown promising results in treating mental and substance use disorders, including major depressive disorder (MDD), treatment-resistant depression (TRD), and alcohol use disorder (AUD).^20^ Studies have shown that PT may reduce SI. Carhart-Harris et al. found a significant reduction in suicidality scores on the QIDS-SR16 1 and 2 weeks post-psilocybin with psychological support in people with TRD. Furthermore, in this study, the suicide item of the Hamilton Depression Rating Scale was significantly decreased 1 week posttreatment, with none showing an increase from baseline.^21^ Carhart-Harris et al.^22^ found a decrease in suicidal ideation from PT compared to escitalopram treatment that was significantly different. Furthermore, Ross et al.^23^ found that PT was associated with a reduction in SI as early as 8 h after treatment, which persisted as long as 6.5 months post-dosing. However, some trials have reported more cases of SI and SA compared to control groups.^24,25^ The mechanism of action of PT remains unclear, although agonist activity on serotonin receptors in the brain, particularly the 5-HT2A receptor, has been implicated.^26^ 5-HT2A activation results in modulation of the amygdala, a key brain region involved in emotional processing, which may account for the therapeutic effects of PT in depression and anxiety.^27^ A previous meta-analysis found that psychedelic therapy had a significant reduction in suicidal behaviors, including SI and SA.^24^ However, this was not specific to psilocybin, and since the completion of the meta-analysis, multiple new clinical trials with control groups investigating PT have been completed and published. Therefore, given these uncertainties and the evolving nature of current evidence, an updated analysis of evidence is warranted.

In this context, we conducted a systematic review and meta-analysis of published randomized clinical trials on the effects of PT on SI, SA, and suicide death outcomes in adults with psychiatric diagnoses.

## Methods

This systematic review was registered on PROSPERO (CRD42023445706) and adhered to the Preferred Reporting Items for Systematic Reviews and Meta-Analyses (PRISMA) 2020 guidelines.^28^

### Search strategy and inclusion and exclusion criteria

MEDLINE, EMBASE, Cochrane, and PsychINFO were searched for randomized controlled trials (RCTs) reporting the effect of psilocybin therapy on suicidal ideation, suicide attempts, or suicide deaths in those with psychiatric disorders, with no limits set on the date of publication. A health sciences librarian (RS) was involved to optimize the search strategy (sTable 1). A manual search was also conducted on Google Scholar to ensure that all relevant articles were included. Studies were included if they met the following criteria: (1) RCT, (2) participants were adults aged >18 years with a diagnosed psychiatric disorder by DSM or ICD criteria, and (3) reported the effect of psilocybin therapy on suicidal ideation, suicide attempts, or suicide deaths, regardless of adjuvant treatment. The suicide outcomes could be primary, secondary, or even reported as adverse events. Studies were excluded if they were not (1) RCT, (2) conducted in adults, or (3) did not report the effect of psilocybin therapy on suicidal ideation, attempts, or deaths. The full search strategy is presented in Table 1.

**Table 1.** Summary of study characteristics.

Study	Sponsor (clinical trial #)	Study design	Sample size (*n*)	Mental health condition investigated	Psilocybin treatment
Bogenschutz et al.^29^ 2022	NYU Lagone Health (NCT02061293)	Randomized controlled trial (diphenhydramine placebo control)	Treatment: 49 Control: 46	Alcohol dependence	Session 1: 25 mg/70 kg single dose Session 2: 25–40 mg/70 kg single dose 4 weeks after first
Carhart-Harris et al.^22^ 2021	Imperial College London (NCT03429075)	Randomized controlled trial (escitalopram control)	Treatment: 30 Control: 29	Major depressive disorder	Two 25 mg single doses 3 weeks apart with daily placebo
Davis et al.^30^ 2020	Johns Hopkins University (NCT03181529)	Randomized controlled trial (waitlist controlled)	Treatment: 13 Control: 11	Major depressive disorder	Session 1: 20 mg/70 kg single dose Session 2: 30 mg/70 kg single dose 1 week after first
Goodwin et al.^25^ 2022	COMPASS Pathways (NCT03775200)	Randomized controlled trial (1 mg psilocybin control)	Treatment: 154 Control: 79	Treatment-resistant major depressive disorder	10 mg or 25 mg single dose
Griffiths et al.^31^ 2016	Johns Hopkins University (NCT00465595)	Randomized controlled trial (low-dose psilocybin control with crossover)	Treatment: 29 Control: 27	Chronic adjustment disorder, dysthymic disorder, generalized anxiety disorder, major depressive disorder	22 mg or 30 mg/70 kg single first dose, then crossover to 1 or 3 mg/70 kg single dose after 5 weeks or vice versa
Raison et al.^32^ 2023	Usona Institute (NCT03866174)	Randomized controlled trial (niacin placebo control)	Treatment: 51 Control: 53	Major depressive disorder	25 mg single dose
Rosenblat et al.^33^ 2024	Brain and Cognition Discovery Foundation (NCT05029466)	Randomized controlled trial (waitlist controlled)	Treatment:16 Control:14	Treatment-resistant major depressive disorder, ADHD, Anxiety disorders, PTSD, OCD, gender dysphoria, personality disorder, and eating disorder	25 mg 1–3 doses
Ross et al.^34^ 2016	NYU Langone Health (NCT00957359)	Randomized controlled trial (niacin control with crossover)	Treatment: 16 Control: 15	Acute stress disorder, generalized anxiety disorder, and adjustment disorder	0.3 mg/kg single dose
Von Rotz et al.^35^ 2023	University of Zurich (NCT03715127)	Randomized controlled trial (placebo pill control)	Treatment: 26 Control: 26	Major depressive disorder	0.215 mg/kg single dose

### Screening

The studies found from the search were imported into Covidence, where screening was completed.^36^ Both title and full-text screening were completed independently in duplicate by two reviewers among authors (SW, GM, NF, SL) with discrepancies resolved through consensus. All references of included studies were screened with the same systematic approach to include all relevant studies.

### Extraction

Three authors (SW, GM, SL) independently extracted relevant data from the included articles and recorded them into a Microsoft Excel spreadsheet designed *a priori*, with discrepancies resolved through consensus. The primary outcomes were suicidal ideation (as recorded on a standardized scale (i.e., the Columbia suicide severity rating scale (C-SSRS)) or as a binary outcome), suicide attempts, and suicide deaths. Suicide behaviors were not considered, given the heterogeneity in definition between studies. If a study included multiple scales assessing SI, the more comprehensive and suicide-specific scale was used. For example, the C-SSRS would be used instead of the suicidal ideation component of another scale. For each RCT, we extracted the standard identifier (PMID or DOI), first author name, year of publication, country of the primary author, if the study was industry-funded or not, number of participants, duration of follow-up, whether suicide-related outcomes were directly measured (i.e., declared in methods or indirectly as an adverse event), demographics (age, sex, ethnicity), comorbid medical or physical conditions, and information regarding the psilocybin and control conditions.

### Quality assessment

Three authors (SW, GM, SL) independently assessed the quality of all included studies using the Cochrane’s Risk of Bias 2 tool.^37^ All discrepancies were resolved through discussion, and the final determination was made by consensus.

### Statistical analysis

All statistical analyses and meta-analyses were performed using Comprehensive Meta-Analysis version 4.^31^

A meta-analysis was performed to calculate a risk ratio (RR) for dichotomous variables or a pooled standardized mean difference (SMD) for continuous variables (where 0.2, 0.5, and 0.8 were used as indicators of small, medium, and large effect sizes, respectively).^39^ A continuity correction of 0.5 was added to both arms when one arm had 0 events when calculating the RR.^40^ Trials with no events in any arms were included for meta-analysis. We used the *I*^2^ statistic to assess heterogeneity, where *I*^2^ > 0.50 was considered substantial.^41,42^ For a conservative estimate of effect size, the random-effects model was used assuming that there was no true homogeneity among included studies.^43^ Forest plots were used to graphically present the significant findings. Funnel plots and Egger’s Test were used to analyze the potential for publication bias.^44^

Subgroup analysis was conducted based on blinding status, risk of bias, study quality, study location, whether suicide outcomes were directly measured, and industry funding status.

## Results

**Figure 1.**
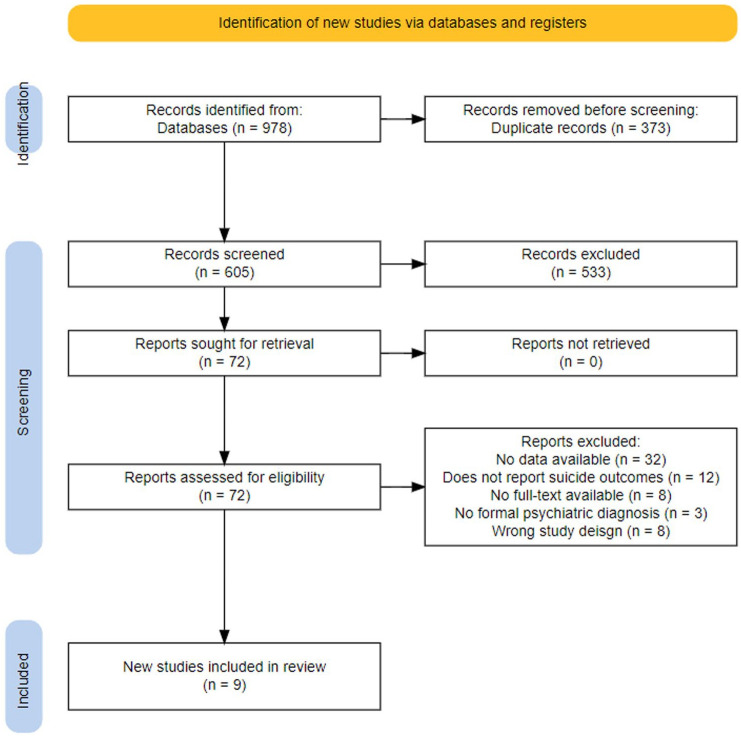
PRISMA diagram of screening of studies for extraction. PRISMA, Preferred reporting items for systematic reviews and meta-analyses.

Six hundred and five abstracts were screened, 72 full-text articles were further examined, and nine articles were extracted after an updated search on July 1, 2024 (Figure 1). This resulted in a total sample size of 593 participants with 335 in the psilocybin therapy arm and 258 in the control arm.

### Study characteristics

Five of the studies were conducted in the United States, one in Canada, one in Switzerland, and one in the United Kingdom. One study was conducted internationally with 10 different locations. Five studies were publicly funded and four were industry sponsored (Table 1). Two studies were published in the 2010s, while the rest were in the 2020s. Psilocybin dosages and frequencies are presented in further detail in Table 1. All studies included psychotherapy or psychological support and required that participants not be using any serotonergic medications. With respect to blinding, only two studies^31,34^ assessed the integrity of blinding.

All participants were diagnosed using the Diagnostic and Statistical Manual for Mental Disorders (DSM). Participants with depression (major depressive, treatment-resistant, and persistent depressive disorder) were observed in 10 studies. Participants with anxiety disorders (generalized anxiety disorder, panic disorder, phobias, social anxiety, and adjustment disorders) were observed in four studies. Bipolar II disorder (in a depressive episode) and alcohol dependence were specifically studied in one study, respectively.

### Suicide attempts and death-related outcomes

No study specifically reported suicide attempts (sTable 3). Suicide deaths were not reported by any of the studies. Three studies specifically reported on “suicidal behaviors” as an adverse event which includes suicidal ideation, attempts, and deaths. The definition of suicidal behavior was from the MedDRA 23.0.^45^ von Rotz et al.^35^ and Raison et al.^32^ reported no suicidal behavior in both psilocybin and control groups. Goodwin et al.^25^ reported three people who exhibited suicidal behavior in the 25 mg psilocybin group, none in the 10 mg group, and none in the 1 mg control group. Due to the limited number of studies, a meta-analysis could not be completed to calculate the risk ratio.

### Suicidal ideation outcomes

Measures of suicidal ideation and the frequencies of suicidal ideation, suicide attempts, and suicide deaths are summarized in sTable 3.

#### Quantitative synthesis

**Figure 2.**
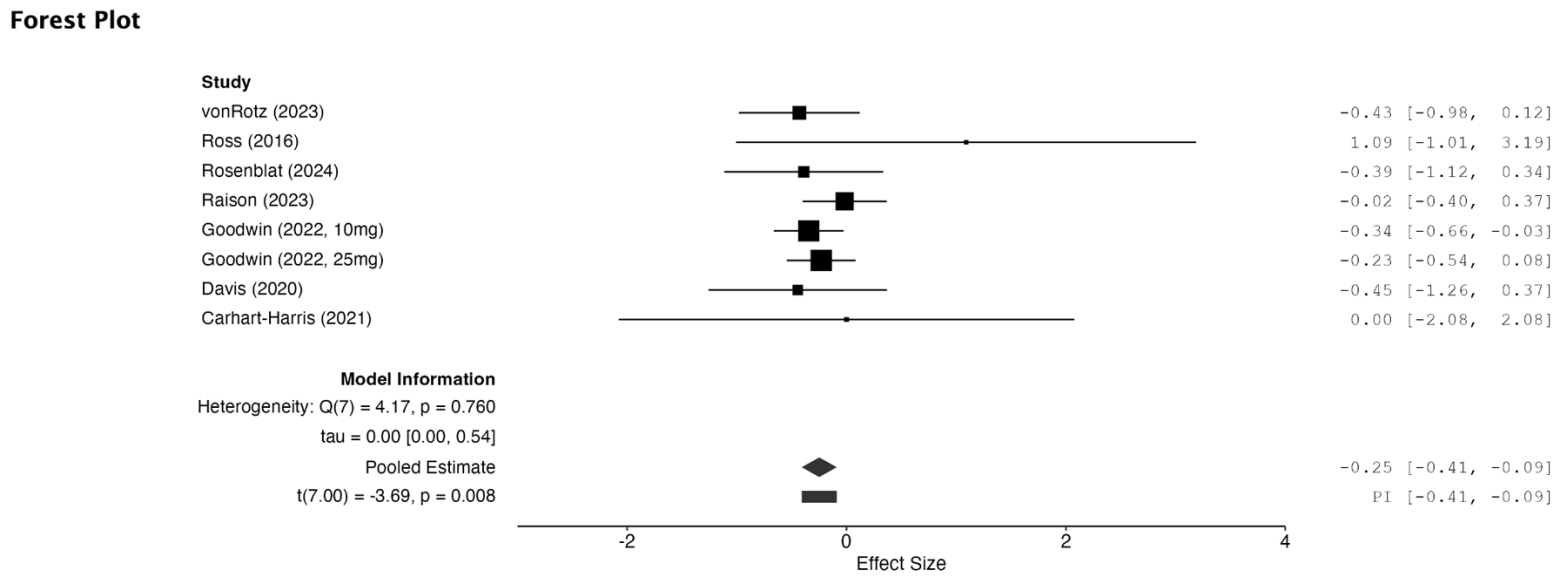
Forest plot of the effect of psilocybin therapy on suicidal ideation compared to control.

Seven RCTs were included in the meta-analysis. Two RCTs were excluded from meta-analysis as the suicide related outcomes data were not available after multiple attempts were made to contact the authors. Patients treated with PT experienced a decrease in SI when compared to those in the control arm of their respective study that had a small and significant effect size (SMD = −0.24, 95% CI −0.42 to −0.06, *p* = 0.008) (Figure 2). Heterogeneity was not significant between studies (*I*^2^ = 0%, *p* = 0.89). For publication bias, funnel plot analysis showed a generally symmetrical plot (sFigure 1). Egger’s test was not significant for publication bias (*p* = 0.67).

#### Subgroup analysis for SI

##### Location

Studies conducted in the United States had a small effect size (SMD = −0.06, 95% CI −0.82 to 0.70, *p* = 0.76, *k* = 3). The effect size in the study by Rosenblat et al.^33^ conducted in Canada was small (SMD = −0.397, 95% CI −1.12 to 0.34) and the effect size in the study by von Rotz et al.^35^ conducted in Switzerland was also small (SMD = −0.43, 95% CI −0.98 to 0.12). There was no significant difference between the effect sizes (*p* = 0.74). All the studies in the United States involved blinding and direct measurements of suicidal ideation.

##### Risk of bias

After removing high-risk-of-bias studies, the remaining studies were all of some concern. There was a small effect size (SMD = −0.089, 95% CI −0.34 to 0.16, *p* = 0.41, *I*^2^ = 0%).

##### Industry sponsored

Studies that were not industry sponsored had a small effect size (SMD = −0.36, 95% CI −0.74 to −0.02, *p* = 0.06, *k* = 5, *I*^2^ = 0%) compared to studies that were not industry sponsored (SMD = −0.22, 95% CI −0.61 to 0.17, *p* = 0.13, *k* = 2, *I*^2^ = 0%). There was no significant difference between the effect sizes (*p* = 0.769).

##### Blinding

Studies with blinding had a statistically significant small effect size (SMD = −0.24, 95% CI −0.42 to −0.06, *p* = 0.02, *k* = 6, *I*^2^ = 0%). Studies with no blinding had a small effect size (SMD = −0.397, 95% CI –1.121 to 0.328, *p* = 0.283, *k* = 1, *I*^2^ = 0%). There was no significant difference between the effect sizes based on blinding (*p* = 0.703). The studies by Ross et al.^34^ and Griffiths et al.^31^ were the only studies that assessed the integrity and effectiveness of their blinding. The study by Ross et al. used a low dose of niacin (250 mg) as a control to preserve the blind, and staff members were able to correctly guess which treatment the participants received in 28/29 cases (97%). In the study by Griffith, all staff approached were able to correctly identify psilocybin use, and they estimated the dose as significantly higher in the high-dose group than in the low-dose group on a 10-point visual analog scale (7.0 ± 0.29 vs 1.7 ± 0.21, *p* < 0.001). It should be noted that in the study by Davis et al.^30^ the blinding was of the clinician raters.

##### Direct measurement of SI compared to indirect

Studies that measured SI directly (using a standardized scale that assesses suicide such as the C-SSRS) had a statistically significant small effect size (SMD = −0.252, 95% CI −0.430 to −0.074, *p* = 0.005, *k* = 5, *I*^2^ = 0%). Studies that did not measure SI directly had a small effect size (SMD = −0.397, 95% CI −1.121 to 0.328, *p* = 0.283, *k* = 1, *I*^2^ = 0%). There was no significant difference between the effect sizes (*p* = 0.703). Rosenblat et al.^33^ used the suicidal ideation component of the MADRS. Ross et al.^34^ used the suicidal ideation component of the BDI. Carhart-Harris et al.^22^ used the Suicidal Ideation Attribution Scale (SIDAS). The remaining studies used the C-SSRS.

##### Dosage

Only single-dose studies had a statistically significant small effect size (SMD = −0.24, 95% CI −0.42 to −0.06, *p* = 0.02, *k* = 5, *I*^2^ = 0%). Two doses as seen in the study by Davis et al. (2021)^30^ had a small effect size (SMD = −0.445, 95% CI −1.247 to 0.386, *p* = 0.284, *k* = 1, *I*^2^ = 0%) and in the study by Rosenblat et al. (2024)^33^ with 1–3 doses had a small effect size (SMD = −0.397, 95% CI −1.121 to 0.328, *p* = 0.283, *k* = 1, *I*^2^ = 0%). There was no significant difference in effect size between dosage groups (*p* = 0.830). It should be noted that the multiple doses had a larger effect size than a single dose, which might potentially be due to the smaller sample sizes in the studies with a higher number of doses.

### Risk of bias and certainty of evidence

**Figure 3.**
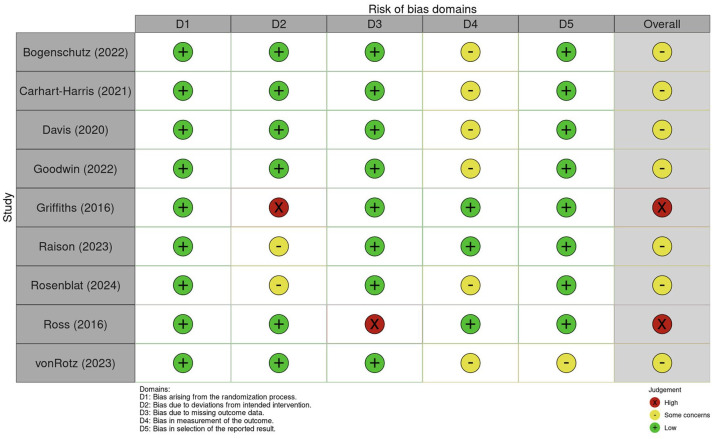
Risk of bias assessment of RCT studies. RCT, randomized controlled trial.

In terms of quality, RCTs were assessed using the Cochrane Risk of Bias Tool 2 and the results are shown in Figure 3. Although blinding was present in all studies except Rosenblat et al. (2024),^33^ Ross et al. (2016)^34^ and Griffiths et al. (2016)^31^ assessed the integrity and effectiveness of blinding based on treatment providers’ guesses, rather than those of the participants.

## Discussion

This meta-analysis found that psilocybin therapy has a small and significant effect size in reducing suicidal ideation when compared to controls (SMD = −0.24, 95% CI −0.42 to −0.06, *p* = 0.008). These findings should be tempered by the findings of the lowest risk of bias study, by Goodwin et al.,^25^ which included active comparison groups and reported higher rates of worsening suicidal ideation that peaked within the first 3 weeks after administration in all groups (6% in 25 mg group, 5% in 10 mg group, and 3% in the 1 mg placebo group). We conducted a Chi-square test and found no statistical difference between the dosage groups (*p* = 0.507). Additionally, out of all nine studies, there was only one suicide death which was concluded by the authors (Griffiths et al., 2016)^31^ to not be related to administration of psilocybin as the participant had received a single 1 mg placebo dose of psilocybin and there was no observable behavioral impairment or adverse sequalae prior to this participant insisting on leaving the study 11 days after the first session. These findings are supported by previous research suggesting that psilocybin therapy very rarely leads to acute or post-acute elevations in suicidal ideation.^24^ Although worsening SI is a rare, individual case reports of worsening SI and completed suicide post-PT have been published. Müller et al. reported a case where a 60-year-old male developed delusions and emotional dysregulation, who died by suicide post-PT. They identified a weak therapeutic alliance that hindered the assessment of the patient’s internal state which reinforced the need for thorough assessments and close follow-up after PT.^46^ Wahba et al. reported worsening SI post-PT that outlasted the acute psychedelic effects due to the patient being a nonresponder and having higher expectations of PT in treating their depression. This report highlighted the importance of managing expectations of patients undergoing PT, given its recent popularity.^47^ As more studies and case reports are published, the impact of PT on suicidal ideation and other suicide-related outcomes will be further expanded upon. Overall, our systematic review and meta-analysis support the findings of previous systematic reviews and meta-analyses that psilocybin therapy can lead to a reduction in suicidal ideation in people with a psychiatric diagnosis.^24^ However, there were insufficient data to determine the effects of psilocybin therapy on suicide attempts and deaths, making suicidal ideation a problematic surrogate outcome.

In general, it can be speculated that the decrease in suicidal ideation after psilocybin therapy is related to the antidepressant effect of this treatment (i.e., improvements in mood and depression-related symptoms would generally have a decrease in suicidal ideation). The specific mechanism of action of psilocybin on suicidal ideation remains unclear. Based on the current literature in the context of psilocybin therapy for depression, it’s biological mechanisms include acting as a serotonin 5-HT(2A) receptor agonist, influences glutamate transmission affecting brain cell regeneration and neuroplasticity, and modulates brain connectivity in the Default Mode Network.^27,48–50^ As a result, the immediate effects of PT result in higher levels of mystical experiences, emotional breakthroughs, and ego dissolution that are associated with greater antidepressant responses.^51^ This is further supported by the findings of reduced experiential avoidance as a putative mechanism underlying PT which has also been found to be associated with the therapeutic response.^52^ However, it should be noted that these mechanisms of action have not been studied in the context of suicide specifically. Ross et al.^23^ hypothesized that the positive impact of PT on hopelessness, demoralization, and its effects on meaning-making in particular can lead to a reduction in SI. Another means of explaining the mechanism of action would be investigating the impact of psilocybin therapy on biomarkers of suicide. Although there are potential biomarkers for suicidal ideation, mainly molecular biomarkers (SAT1, CRP, IL-6, and BCL2), to the best of our knowledge, no trial specifically investigated associations between suicidal ideation and biomarkers.^53–55^ Future trials may consider integrating biomarkers to advance the understanding of the biological mechanism of action of psilocybin therapy on suicidal ideation.

### Limitations

The results of this review need to be interpreted with caution, given the small sample sizes in included studies, which can exaggerate the effect sizes of psilocybin therapy. There is also the influence of industry funding, which may influence the publication of positive findings; however, our subgroup analysis found no significant difference between the effect sizes in industry- and nonindustry-funded studies. There is also a high probability of functional unblinding in psilocybin therapy trials, given the acute psychedelic effects of the drug. Two studies^31,34^ demonstrated rigor by assessing the integrity of blinding by having clinician raters guess whether participants received psilocybin or an active placebo. Ross et al.^34^ found that raters guessed correctly in 97% of participants, which suggested potential evidence of functional unblinding in psilocybin studies. Future studies should consider the use of appropriate active comparators to preserve blinding and robustly assess the integrity of the blind. Furthermore, there is limited long-term follow-up data on psilocybin therapy on suicide outcomes over an extended time period. In the meta-analysis by Zeifman et al.^24^, a decrease in SI was observed following PT although this effect was not consistent across all time points. This speaks to the novelty of this field of research, and future follow-up studies would be beneficial. In all included studies, the reporting of suicide-related outcomes being classified as “serious” or “other” was based on the clinician’s subjective assessment, which may have influenced the reporting. Standardization of suicide outcome reporting using guidelines such as the Office for Human Research Protections (OHRP) Guidance 2007 would allow for better comparison of suicide related outcomes and other adverse events.^56^ Additionally, our review found that there was significant heterogeneity in the administration of PT as studies varied by dosing and type or amount of psychological support provided. Another limitation is the lack of information on the impact of psilocybin on suicidal ideation, attempts, and deaths among various psychiatric conditions. In our review, five studies were in MDD populations, one in alcohol dependency, and three were mixed populations that did not specify outcomes per population group. It is well established that different psychiatric disorders have different risks for suicide and suicide-related outcomes.^57,58^ Therefore, future studies should report more specific adverse events per different psychiatric diagnoses to allow for a better understanding of the effect of psilocybin on suicide-related outcomes. Lastly, a common exclusion criterion in psilocybin trials has been participants experiencing severe suicidal ideation at baseline or with a history of suicide attempts, which would limit the applicability of psilocybin therapy in populations with more chronic or elevated risks of harm to self. This could lead to a potential for floor effects, which may have influenced the results of the meta-analysis. If individuals with high degrees of SI were excluded, it may be possible that many participants had baseline SI scores of zero, which may lead to little room for measuring improvement. As such, changes in the mean level of SI may not accurately reflect treatment effects. Given these limitations of the current literature, the results of this meta-analysis must be interpreted with a degree of caution. Future studies would benefit from larger sample sizes with different psychiatric diagnoses investigated in different settings to best address the limitations of the current literature.

## Conclusion

Psilocybin therapy has shown initial promising results in the treatment of depressive disorders and potentially other psychiatric diagnoses. There is emerging clinical trial evidence of decreased suicidal ideation from psilocybin therapy. This systematic review and meta-analysis adds to the current literature by reporting a significantly greater reduction in suicidal ideation among participants receiving psilocybin therapy compared to control conditions. Future studies would benefit from long-term follow-up data on suicidal ideation to determine the duration of the effect of psilocybin on suicidal ideation. Furthermore, future studies will benefit from larger and more diverse sample sizes as well as innovative efforts to preserve blinding to provide a more robust assessment of psilocybin therapy’s effects on suicidal ideation and other suicide-related outcomes.

## Supplemental Material

sj-docx-1-tpp-10.1177_20451253251372449 – Supplemental material for Effect of psilocybin therapy on suicidal ideation, attempts, and deaths in people with psychiatric diagnoses: a systematic review and meta-analysis Supplemental material, sj-docx-1-tpp-10.1177_20451253251372449 for Effect of psilocybin therapy on suicidal ideation, attempts, and deaths in people with psychiatric diagnoses: a systematic review and meta-analysis by Stanley Wong, Gray Meckling, Nicholas Fabiano, Sanghun Lee, Brett D. M. Jones, Risa Shorr, Aroldo Dargel, Alan K. Davis, Jess G. Fiedorowicz, Marco Solmi, Joshua D. Rosenblat, Benoit H. Mulsant, Daniel M. Blumberger and Muhammad Ishrat Husain in Therapeutic Advances in Psychopharmacology

## Declarations

**Ethics approval and consent to participate:** Research ethics board approval is not required for systematic reviews using publicly available information under Canada’s Tri-council Policy Statement.

**Consent for publication:** Not required for systematic reviews.

**Availability of data and materials:** Data can be requested from corresponding author.
