# Supplementary material for: Effect of psilocybin therapy on suicidal ideation, attempts, and deaths in people with psychiatric diagnoses: a systematic review and meta-analysis
Source: Ther Adv Psychopharmacol. 2025 Sep 7;15:20451253251372449. doi: 10.1177/20451253251372449 (PMC12417673; doi:10.1177/20451253251372449)
Supplement: sj-docx-1-tpp-10.1177_20451253251372449 – Supplemental material for Effect of psilocybin therapy on suicidal ideation, attempts, and deaths in people with psychiatric diagnoses: a systematic review and meta-analysis [file sj-docx-1-tpp-10.1177_20451253251372449.docx]

**Supplemental Material**

**Title**: Effect of psilocybin therapy on suicidal ideation, attempts, and deaths in people with psychiatric diagnoses: a systematic review and meta-analysis

**Authors**: Stanley Wong MD^1^, Gray Meckling MD^2^, Nicholas Fabiano MD^2^, Sanghun Lee, Brett D.M. Jones MD^1,3^, Risa Shorr MLS^4^, Aroldo Dargel MD PhD^2,5,6,^, Alan K. Davis^7,8, 9^**,** Jess G. Fiedorowicz MD PhD^2,5,6,10-^, Marco Solmi MD PhD^2,5,6,10,11^, Joshua D. Rosenblat MD^1,12^, Benoit H. Mulsant MD^1,3^, Daniel M. Blumberger MD^1,3,13^, M. Ishrat Husain MBBS, MD (Res)^1,3^

1. Department of Psychiatry, University of Toronto, Toronto, Ontario, Canada
2. Department of Psychiatry, University of Ottawa, Ottawa, Ontario, Canada
3. Campbell Family Mental Health Research Institute, Centre for Addiction and Mental Health, Toronto, Ontario, Canada
4. Library Services, The Ottawa Hospital, Ottawa, ON, Canada
5. Department of Mental Health, The Ottawa Hospital, Ottawa, ON, Canada
6. Ottawa Hospital Research Institute (OHRI), Ottawa, ON, Canada
7. Center for Psychedelic Drug Research and Education, College of Social Work, The Ohio State University, Columbus, OH, USA
8. Center for Psychedelic and Consciousness Research, School of Medicine, Johns Hopkins University, Baltimore, MD, USA
9. Department of Internal Medicine, Ohio State University, Columbus, OH 43210
10. School of Epidemiology and Public Health, Faculty of Medicine, University of Ottawa, Ottawa, ON, Canada
11. Department of Child and Adolescent Psychiatry, Charité Universitätsmedizin, Berlin, Germany
12. Mood Disorder Pharmacology Unit, University Health Network, Toronto, Ontario, Canada
13. Temerty Centre for Therapeutic Brain Intervention, CAMH, Toronto

Stanley Wong (Corresponding author): sta.wong@mail.utoronto.ca

**Funding:** None

**Data Availability:** Data can be requested from corresponding author

**Ethics:** Research ethics board approval is not required for systematic reviews using publicly available information under Canada’s Tri-council Policy Statement

**Acknowledgements:** None

**Disclosures:**

**MIH** receives research support from the Brain and Behavior Research Foundation, Canadian Institutes of Health Research (CIHR), CAMH Foundation, Grand Challenges Canada, the PSI Foundation, and the University of Toronto. He has provided consultancy to Mindset Pharma, PsychEd Therapeutics, and Wake Network. He has led contracted research for Compass Pathfinder Limited.

**DMB** receives research support from CIHR, NIH, Brain Canada and the Temerty Family through the CAMH Foundation and the Campbell Family Research Institute. He received research support and in-kind equipment support for an investigator-initiated study from Brainsway Ltd. He was the site principal investigator for three sponsor-initiated studies for Brainsway Ltd. He also receives in-kind equipment support from Magventure for investigator-initiated research. He received medication supplies for an investigator-initiated trial from Indivior. He is a Scientific Advisor for Sooma Oy Medical.

**AKD** is supported by the Center for Psychedelic Drug Research and Education, funded by anonymous private donors. AKD is also supported by the Johns Hopkins Center for Psychedelic and Consciousness Research, funded by private philanthropic funding from Tim Ferriss, Matt Mullenweg, Craig Nerenberg, Blake Mycoskie, and the Steven and Alexandra Cohen Foundation. AKD is also supported by the Comprehensive Cancer Center at Ohio State University. The funding sources had no role in the study, data analysis, interpretation, or communication of findings. AKD is also a board member of Source Research Foundation. This organization was not involved in this research.

**JDR** has received research grant support from the Canadian Institute of Health Research (CIHR), Physician Services Inc (PSI) Foundation, Labatt Brain Health Network, Brain and Cognition Discovery Foundation (BCDF), Canadian Cancer Society, Canadian Psychiatric Association, Academic Scholars Award, American Psychiatric Association, American Society of Psychopharmacology, University of Toronto, University Health Network Centre for Mental Health, Joseph M. West Family Memorial Fund and Timeposters Fellowship and industry funding for speaker/consultation/research fees from iGan, Boehringer Ingelheim, Janssen, Allergan, Lundbeck, Sunovion and COMPASS. He was previously the Chief Medical and Scientific Officer of Braxia Scientific and the medical director of the Canadian Rapid Treatment Centre of Excellence (Braxia Health).

**BHM** holds and receives support from the Labatt Family Chair in Biology of Depression in Late-Life Adults at the University of Toronto. He currently receives or has received with the past five years research support from Brain Canada, the Canadian Institutes of Health Research, the CAMH Foundation, the Patient-Centered Outcomes Research Institute (PCORI), the US National Institute of Health (NIH), Capital Solution Design LLC (software used in a study founded by CAMH Foundation), Eli Lilly (medications for a NIH-funded clinical trial), HAPPYneuron (software used in a study founded by Brain Canada), and Pfizer (medications for a NIH-funded clinical trial). He has also been an unpaid consultant to Myriad Neuroscience.

**MS** has received honoraria/has been a consultant for AbbVie, Angelini, Lundbeck, Otsuka.

Table of Contents

**Table of Contents**

sTable 1. PRISMA 2020 Checklist

sTable 2. Search Strategy

sFigure 1. Funnel plot assessing for publication bias in the effect of psilocybin therapy on suicidal ideation compared to control

**sTable 1. PRISMA 2020 Checklist**

| **Section and Topic** | **Item #** | **Checklist item** | **Location where item is reported** |
| --- | --- | --- | --- |
| **TITLE** | | |  |
| Title | 1 | Identify the report as a systematic review. | Title page |
| **ABSTRACT** | | |  |
| Abstract | 2 | See the PRISMA 2020 for Abstracts checklist. | Page 2 |
| **INTRODUCTION** | | |  |
| Rationale | 3 | Describe the rationale for the review in the context of existing knowledge. | Page 3-4 |
| Objectives | 4 | Provide an explicit statement of the objective(s) or question(s) the review addresses. | Page 3-4 |
| **METHODS** | | |  |
| Eligibility criteria | 5 | Specify the inclusion and exclusion criteria for the review and how studies were grouped for the syntheses. | Page 4-5 |
| Information sources | 6 | Specify all databases, registers, websites, organisations, reference lists and other sources searched or consulted to identify studies. Specify the date when each source was last searched or consulted. | Page 4-5 |
| Search strategy | 7 | Present the full search strategies for all databases, registers and websites, including any filters and limits used. | Page 4-5 |
| Selection process | 8 | Specify the methods used to decide whether a study met the inclusion criteria of the review, including how many reviewers screened each record and each report retrieved, whether they worked independently, and if applicable, details of automation tools used in the process. | Page 4-5 |
| Data collection process | 9 | Specify the methods used to collect data from reports, including how many reviewers collected data from each report, whether they worked independently, any processes for obtaining or confirming data from study investigators, and if applicable, details of automation tools used in the process. | Page 4-5 |
| Data items | 10a | List and define all outcomes for which data were sought. Specify whether all results that were compatible with each outcome domain in each study were sought (e.g. for all measures, time points, analyses), and if not, the methods used to decide which results to collect. | Page 4-5 |
|  | 10b | List and define all other variables for which data were sought (e.g. participant and intervention characteristics, funding sources). Describe any assumptions made about any missing or unclear information. | Page 4-5 |
| Study risk of bias assessment | 11 | Specify the methods used to assess risk of bias in the included studies, including details of the tool(s) used, how many reviewers assessed each study and whether they worked independently, and if applicable, details of automation tools used in the process. | Page 4-5 |
| Effect measures | 12 | Specify for each outcome the effect measure(s) (e.g. risk ratio, mean difference) used in the synthesis or presentation of results. | Page 4-5 |
| Synthesis methods | 13a | Describe the processes used to decide which studies were eligible for each synthesis (e.g. tabulating the study intervention characteristics and comparing against the planned groups for each synthesis (item #5)). | Page 4-5 |
|  | 13b | Describe any methods required to prepare the data for presentation or synthesis, such as handling of missing summary statistics, or data conversions. | Page 4-5 |
|  | 13c | Describe any methods used to tabulate or visually display results of individual studies and syntheses. | Page 4-5 |
|  | 13d | Describe any methods used to synthesize results and provide a rationale for the choice(s). If meta-analysis was performed, describe the model(s), method(s) to identify the presence and extent of statistical heterogeneity, and software package(s) used. | Page 4-5 |
|  | 13e | Describe any methods used to explore possible causes of heterogeneity among study results (e.g. subgroup analysis, meta-regression). | Page 4-5 |
|  | 13f | Describe any sensitivity analyses conducted to assess robustness of the synthesized results. | Page 4-5 |
| Reporting bias assessment | 14 | Describe any methods used to assess risk of bias due to missing results in a synthesis (arising from reporting biases). | Page 4-5 |
| Certainty assessment | 15 | Describe any methods used to assess certainty (or confidence) in the body of evidence for an outcome. | Page 4-5 |
| **RESULTS** | | |  |
| Study selection | 16a | Describe the results of the search and selection process, from the number of records identified in the search to the number of studies included in the review, ideally using a flow diagram. | 4-5 |
|  | 16b | Cite studies that might appear to meet the inclusion criteria, but which were excluded, and explain why they were excluded. | N/A |
| Study characteristics | 17 | Cite each included study and present its characteristics. | Table 1 |
| Risk of bias in studies | 18 | Present assessments of risk of bias for each included study. | Figure 3 |
| Results of individual studies | 19 | For all outcomes, present, for each study: (a) summary statistics for each group (where appropriate) and (b) an effect estimate and its precision (e.g. confidence/credible interval), ideally using structured tables or plots. | sTable 3 |
| Results of syntheses | 20a | For each synthesis, briefly summarise the characteristics and risk of bias among contributing studies. | Figure 3 |
|  | 20b | Present results of all statistical syntheses conducted. If meta-analysis was done, present for each the summary estimate and its precision (e.g. confidence/credible interval) and measures of statistical heterogeneity. If comparing groups, describe the direction of the effect. | Figure 2 |
|  | 20c | Present results of all investigations of possible causes of heterogeneity among study results. | Page 7 |
|  | 20d | Present results of all sensitivity analyses conducted to assess the robustness of the synthesized results. | N/A |
| Reporting biases | 21 | Present assessments of risk of bias due to missing results (arising from reporting biases) for each synthesis assessed. | Figure 3 |
| Certainty of evidence | 22 | Present assessments of certainty (or confidence) in the body of evidence for each outcome assessed. | Page 7 |
| **DISCUSSION** | | |  |
| Discussion | 23a | Provide a general interpretation of the results in the context of other evidence. | Page 9-11 |
|  | 23b | Discuss any limitations of the evidence included in the review. | Page 9-11 |
|  | 23c | Discuss any limitations of the review processes used. | Page 9-11 |
|  | 23d | Discuss implications of the results for practice, policy, and future research. | Page 9-11 |
| **OTHER INFORMATION** | | |  |
| Registration and protocol | 24a | Provide registration information for the review, including register name and registration number, or state that the review was not registered. | Page 4 |
|  | 24b | Indicate where the review protocol can be accessed, or state that a protocol was not prepared. | Page 4 |
|  | 24c | Describe and explain any amendments to information provided at registration or in the protocol. | N/A |
| Support | 25 | Describe sources of financial or non-financial support for the review, and the role of the funders or sponsors in the review. | Title page |
| Competing interests | 26 | Declare any competing interests of review authors. | Page 1 |
| Availability of data, code and other materials | 27 | Report which of the following are publicly available and where they can be found: template data collection forms; data extracted from included studies; data used for all analyses; analytic code; any other materials used in the review. | Title page |

*From:*  Page MJ, McKenzie JE, Bossuyt PM, Boutron I, Hoffmann TC, Mulrow CD, et al. The PRISMA 2020 statement: an updated guideline for reporting systematic reviews. BMJ 2021;372:n71. doi: 10.1136/bmj.n71. This work is licensed under CC BY 4.0. To view a copy of this license, visit<https://creativecommons.org/licenses/by/4.0/>

**sTable 2. Search Strategy**

| Ovid MEDLINE(R) ALL <1946 to July 12, 2023>    1 Psilocybin/ 1101  2 Psilocybin*.tw,kf. 1388  3 magic mushroom*.tw,kf. 134  4 1 or 2 or 3 1725  5 exp Mental Disorders/ or exp Substance-Related Disorders/ or Behavior, Addictive/ 1466954  6 self-injurious behavior/ or exp suicide/ 80861  7 suicid*.tw,kf. 96610  8 (self harm or self injur*).tw,kf. 13844  9 Depression/ 150775  10 exp Depressive Disorder/ 121881  11 mood disorders/ 15925  12 (depress* or mood disorder* or bipolar or psychosis or psychotic or addict* or substance abuse*).tw,kf. 770821  13 (alcohol* adj2 disorder*).tw,kf. 20121  14 exp Anxiety Disorders/ 90420  15 Anxiety/ 106698  16 anxiety.tw,kf. 263196  17 Psychiatr*.tw,kf. 289132  18 (mental adj2 (disease* or disorder* or illness*)).tw,kf. 106870  19 or/6-18 1294352  20 4 and 19 773  21 randomized controlled trial.pt. 596097  22 (random* or placebo).tw. or trial.ti. 1587857  23 controlled clinical trial.pt. 95362  24 control group.tw,kf. 506518  25 prospective* trial*.tw. or Trial registration.tw,kf. 113999  26 21 or 22 or 23 or 24 or 25 2134506  27 exp animals/ not exp humans/ 5137253  28 26 not 27 1898619  29 20 and 28 181  Embase Classic+Embase <1947 to 2023 July 12>    1 psilocybine/ 2824  2 Psilocybin*.tw. 1779  3 magic mushroom*.tw. 184  4 or/1-3 3077  5 exp mental disease/ 2809668  6 exp drug dependence/ 296203  7 suicid*.tw. 126475  8 (self harm or self injur*).tw. 17407  9 depress*.tw. 796713  10 (depress* or mood disorder* or bipolar or psychosis or psychotic or addict* or substance abuse*).tw. 1089912  11 (alcohol* adj2 disorder*).tw. 30108  12 psychiatr*.tw. 417985  13 anxiety.tw. 377095  14 (mental adj2 (disease* or disorder* or illness*)).tw. 129853  15 or/5-14 3423594  16 4 and 15 2035  17 random*.tw. or placebo*.mp. or double-blind*.tw. or trial.ti. 2401192  18 control group*.tw. 840963  19 17 or 18 2997124  20 (exp animal/ or animal experiment/ or nonhuman/) not exp human/ 8086930  21 19 not 20 2579583  22 16 and 21 398  EBM Reviews - Cochrane Central Register of Controlled Trials <June 2023>    1 Psilocybin/ 146  2 Psilocybin*.tw,kw. 289  3 magic mushroom*.tw,kw. 10  4 1 or 2 or 3 295  5 exp Mental Disorders/ or exp Substance-Related Disorders/ or Behavior, Addictive/ 98964  6 self-injurious behavior/ or exp suicide/ 2244  7 suicid*.tw,kf. 6875  8 (self harm or self injur*).tw,kw. 1214  9 Depression/ 18139  10 exp Depressive Disorder/ 15070  11 mood disorders/ 1017  12 (depress* or mood disorder* or bipolar or psychosis or psychotic or addict* or substance abuse*).tw,kw. 126360  13 (alcohol* adj2 disorder*).tw,kw. 3253  14 exp Anxiety Disorders/ 9097  15 Anxiety/ 12396  16 anxiety.tw,kw. 65610  17 Psychiatr*.tw,kw. 24951  18 (mental adj2 (disease* or disorder* or illness*)).tw,kw. 17415  19 or/6-18 180670  20 4 and 19 206  APA PsycInfo <1806 to July Week 1 2023>    1 psilocybin/ 430  2 psilocybin*.tw. 828  3 1 or 2 836  4 (double-blind or random* or control or trial).tw. 745372  5 3 and 4 193 |
| --- |

**sTable 3. Suicide outcomes of included studies**

| Study | SI scale | Score changes : mean (SD) | Suicidal ideation events reported (n) | Suicidal behavior  (n) | Suicide attempts (n) | Suicide death (n) |
| --- | --- | --- | --- | --- | --- | --- |
| Bogenschutz 2022 | None | Not applicable  Note: One participant assigned to the psilocybin group reported passive SI for 15 minutes during a medication session that resolved without sequelae. | Psilocybin: 1  Control: 1 | Not reported | Not reported | Not reported |
| Carhart-Harris 2021 | SIDAS (pre psilocybin and at 6 weeks follow-up) | Not available  Note: On QIDS-SR-16, there was a noted mean change with IQR of the following:  Psilocybin:0 (-5 to 0; n = 30)  Escitalopram: 0 (-4 to 2; n = 29) | Not available | Not reported | Not reported | Not reported |
| Davis 2021 | C-SSRS (taken at every visit prior to psilocybin up to at 8 and 16 weeks follow-up) | Psilocybin  Pre: 1.2 (1.2)  Post: 0.2 (0.4)  Control  Pre: 1.3 (1.3)  Post: 0.5 (0.9) | Not reported | Not reported | Not reported | Not reported |
| Goodwin 2022 | C-SSRS (taken prior to psilocybin and at 3 up to 12 weeks follow-up) | Psilocybin 25 mg  Pre: 0.35 (0.64)  Post: 0.53 (1.06)  Psilocybin 10 mg  Pre: 0.45 (0.70)  Post: 0.59 (0.88)  Control (Psilocybin 1 mg)  Pre: 0.28 (0.53)  Post: 0.33 (0.61) | Psilocybin: 2  Control: 0 | Psilocybin: 3  Control: 0 | Not reported | Not reported |
| Griffiths 2016 | BDI (taken prior to psilocybin , 5 weeks after session 1, 5 weeks after session 2, and at 6 months follow-up) | Psilocybin  Pre: 1.20 (0.45)  Post: 0.50 (1.00) | Not reported | Not reported | Not reported | One instance of a completed suicide with low-dose psilocybin not found to be related to psilocybin |
| Raison 2023 | C-SSRS (taken prior to psilocybin and at 2, 8, 15, 29, and 43 days after dosing) | Psilocybin  Pre: 0.56 (0.67)  Post: 0.29 (0.61)  Control  Pre: 0.39 (0.65)  Post: 0.30 (0.59) | Psilocybin: 1  Control: 5 | Psilocybin: 0  Control: 0 | Not reported | Not reported |
| Rosenblat 2024 | MADRS SI item (taken prior to psilocybin and up to at 24 weeks follow-up) | Psilocybin (immediate)  Pre:2.25 (1.84)  Post:1.94 (2.02)  Control (delayed)  Pre:1.62 (1.45)  Post:1.23 (1.48) | Psilocybin: 2  Control:0 | Not reported | Not reported | Not reported |
| Ross 2016 | BDI (taken prior to psilocybin and at each follow-up up to 26 weeks post second dose) | Psilocybin  Pre: 1.00  Post: 0.33  Control: Not available  Note: In a secondary analysis of this study by Ross et al. (2021), they found a reduction in SI that was significant compared to baseline even at 6.5 months follow-up. | Not applicable | Not reported | Not reported | Not reported |
| vonRotz 2023 | C-SSRS (taken prior to psilocybin and at each follow-up up to 14 days post treatment) | Psilocybin  Pre: 0.5 (0.76)  Post: 0.15 (0.37)  Control  Pre: 0.54 (0.81)  Post: 0.46 (0.95) | Not applicable | Psilocybin: 0  Control: 0 | Not reported | Not reported |

C-SSRS = Columbia-suicide severity rating scale, QIDS-SR16 = Quick inventory of depressive symptoms-self reported, HAM-D = Hamilton rating scale for depression, SIDAS = Suicidal ideation attributes scale, BDI = Beck’s depression inventory, MADRS = Montgomery-Asberg Depression Scale


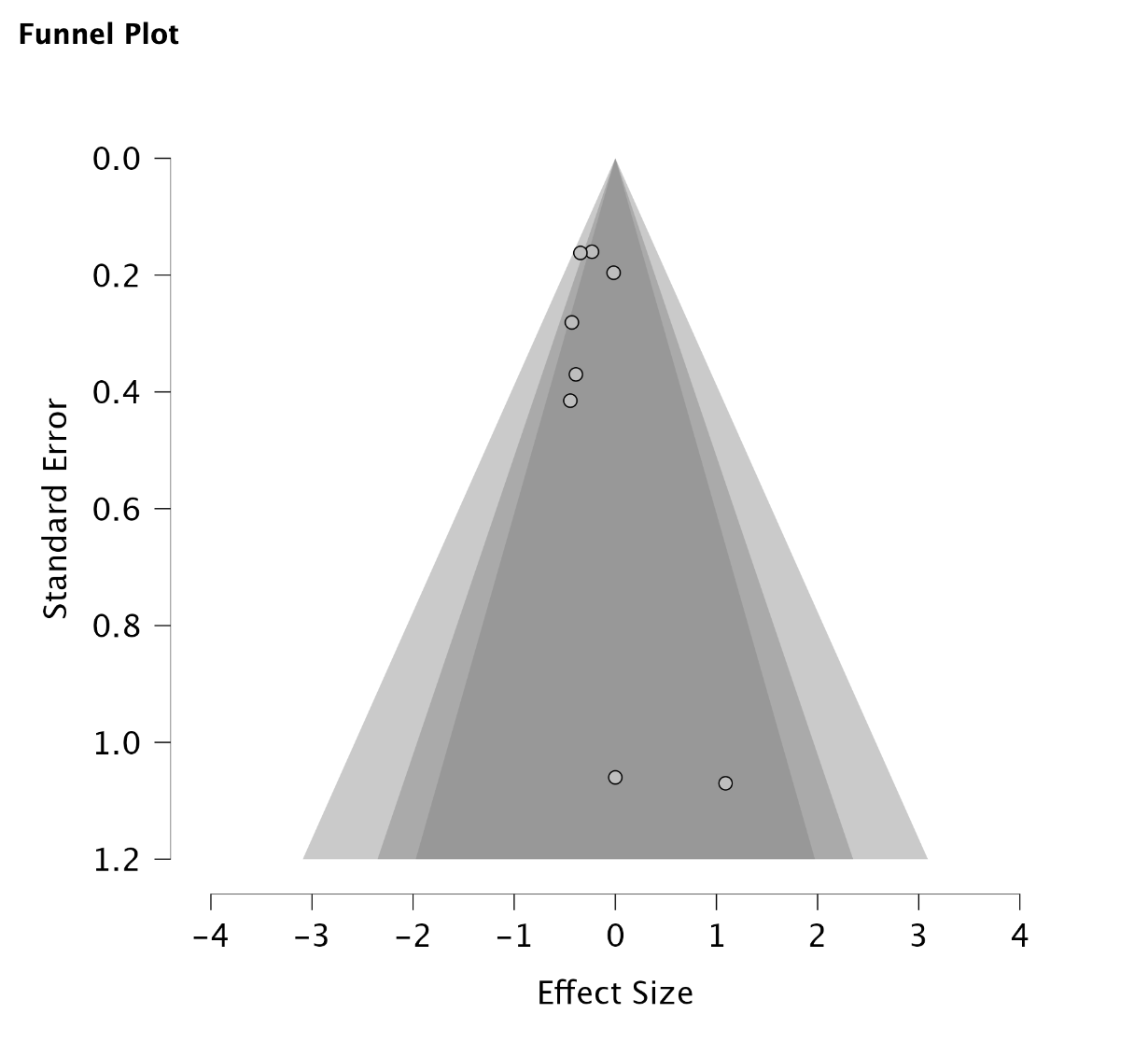


sFigure 1. Funnel plot assessing for publication bias in the effect of psilocybin therapy on suicidal ideation compared to control
